# Health system barriers to HPV vaccination in marginalized Roma communities in Slovakia

**DOI:** 10.1093/eurpub/ckac129.186

**Published:** 2022-10-25

**Authors:** D Filakovska Bobakova, Z Dankulincova Veselska, M Halanova, P Jarcuska

**Affiliations:** 1 Department of Health Psychology and Research Methodology, PJ Safarik University, Kosice, Slovakia; 2 Department of Epidemiology, PJ Safarik University, Kosice, Slovakia; 3 Department of Infectology and Travel Medicine, PJ Safarik University, Kosice, Slovakia

## Abstract

**Background:**

People from marginalized Roma communities often experience poverty, limited access to education, employment, housing, and health care (HC). The aim of the study was to explore the perceptions of people from marginalized Roma communities and health professionals regarding health system (HS) barriers to HPV vaccination.

**Methods:**

A qualitative study was conducted in the Kosice region as a part of the RIVER-EU project. Semi-structured interviews with marginalized Roma parents (N = 18), children (N = 15), and health professionals (N = 18) were audio-recorded and thematic analysis of the transcripts was performed in MAXQDA.

**Findings:**

Four main themes were identified regarding HS barriers: 1. Lack of information (lack of culturally and linguistically appropriate information, lack of information provision from HC providers, unreliable and conflicting information on the internet), 2. Restricted access to HC providers (lack of capacities, work overload, long wait in the waiting room, distance, traffic connection), 3. Financial and organizational barriers (limited coverage of vaccination expenses from health insurance, picking up prescribed vaccines in a pharmacy by parents, parental consent), 4. Attitudes and behaviours of HC providers (neglect of care, double standard, inappropriate behaviour and communication, prejudices, racism).

**Conclusions:**

The reasoning and perception of several barriers to HPV vaccination differ among groups of respondents. Nevertheless, HPV is not viewed as a priority by both - marginalized Roma and health professionals. HS fails to reach marginalized Roma with appropriate information about HPV and HPV vaccination. Moreover, the lack of capacities and motivation of HC providers to address these topics lead to a lack of awareness. Organization and health insurance coverage of vaccination pose additional barriers to HPV vaccination.

